# The Evolution of *Vicia ramuliflora* (Fabaceae) at Tetraploid and Diploid Levels Revealed with FISH and RAPD

**DOI:** 10.1371/journal.pone.0170695

**Published:** 2017-01-30

**Authors:** Ying Han, Yuan Liu, Haoyou Wang, Xiangjun Liu

**Affiliations:** 1 School of Life Science and Engineering, Southwest University of Science and Technology, Mianyang, China; 2 College of Life Sciences, Tianjin Normal University, Tianjin, China; 3 School of life science and technology, Harbin Normal University, Harbin, China; National Cheng Kung University, TAIWAN

## Abstract

*Vicia ramuliflora* L. is a widely distributed species in Eurasia with high economic value. For past 200 years, it has evolved a tetraploid cytotype and new subspecies at the diploid level. Based on taxonomy, cytogeography and other lines of evidence, previous studies have provided valuable information about the evolution of *V*. *ramuliflora* ploidy level, but due to the limited resolution of traditional methods, important questions remain. In this study, fluorescence in situ hybridization (FISH) and random amplified polymorphic DNA (RAPD) were used to analyze the evolution of *V*. *ramuliflora* at the diploid and tetraploid levels. Our aim was to reveal the genomic constitution and parents of the tetraploid *V*. *ramuliflora* and the relationships among diploid *V*. *ramuliflora* populations. Our study showed that the tetraploid cytotype of *V*. *ramuliflora* at Changbai Mountains (M) has identical 18S and 5S rDNA distribution patterns with the diploid Hengdaohezi population (B) and the diploid Dailing population (H). However, UPGMA clustering, Neighbor-Joining clustering and principal coordinates analysis based on RAPD showed that the tetraploid cytotype (M) has more close relationships with Qianshan diploid population T. Based on our results and the fact that interspecific hybridization among *Vicia* species is very difficult, we think that the tetraploid *V*. *ramuliflora* is an autotetraploid and its genomic origin still needs further study. In addition, our study also found that Qianshan diploid population (T) had evolved distinct new traits compared with other diploid populations, which hints that *V*. *ramuliflora* evolved further at diploid level. We suggest that diploid population T be re-classified as a new subspecies.

## Introduction

*Vicia ramuliflora* (Maxim.) Ohwi belongs to the family Fabaceae, tribe Fabeae Rchb. ex Kitt., syn. Vicieae Bronn., genus *Vicia* L. and sect. Vicilla (Schur) Aschers. et Graebn. ex Kupicha. It is a perennial herbaceous species that is widely distributed in Eurasia, especially in East Asia, including Japan, Siberia, Mongolia, China, and Korea. The species of the genus *Vicia* are considered important economically, because they provide energy and protein for livestock [1,2]. They can either be grazed as fresh forage [3] or can be cut and preserved as hay or silage [1,2]. Moreover, they also can be used for green manure [4]. Many *Vicia* species including *V*. *ramuliflora* also are important as herbaceous medicinals [5]. Cytotaxonomic studies [6,7,8] showed that *V*. *ramuliflora* and its closely related *Vicia* species only included diploid (2n = 2x = 12) cytotype in Japan, Russia and Korean Peninsula, but evolved diploid (2n = 2x = 12) and tetraploid (2n = 2x = 24) cytotypes in China. The tetraploid cytotypes are discontinuously distributed in the Changbai Mountains (Northeast China) and Huangshan Mountains, Tianmu Mountains and Lu Mountains (Easten China). In Northeast China, the diploid form of *V*. *ramuliflora* is widespread. However, its conspecific tetraploid counterpart is only endemic to the margins of forests and subalpine meadows of Changbai Mountains at altitudes above 1500–2000 m. Previous fieldwork [7] indicated that the population of the tetraploid form was well-developed, consisting of thousands of individuals. However, when tetraploid individuals of *V*. *ramuliflora* were transplanted to lower altitude regions, their sexual reproduction became abnormal (i.e. the flower buds fall earlier than usual and therefore do not set fruit). Li *et al*. [7] found that diploid and tetraploid *V*. *ramuliflora* populations showed similar karyotypes and had no distinct cytological genetic markers. Hence, the genome components, evolutionary relationships and likely diploid parents of these tetraploid populations could not be drawn out through classical karyotype data and conventional crossing experiments.

Interestingly, it appears that *V*. *ramuliflora* not only evolved and formed the tetraploid genotype to adapt to harsher environmental conditions, but also further evolved at the level of diploid as well. Various authors have reported different opinions on the taxonomic designations of various populations within *V*. *ramuliflora* (Table 1). Their different opinions are mainly focused on the taxonomic status of the diploid Qianshan population (T) and the tetraploid Changbaishan population (M). Geographically, population T occupies a limited geographical area, the Liaotung Peninsula, which is surrounded by sea (to the northeast is the neighboring Changbai Mountains with the Qianshan Mountains running through the middle). Fu & Chen (1976) thought that population T and population M should be treated as two forms: *V*. *ramuliflora* f. *abbreviata* and *V*. *ramuliflora* f. *chianschanensis* [9]. Xia (1996) suggested that the population T should be partitioned as an independent species and population M should be treated as one form of *V*. *ramuliflora* [10]. By contrast, Li *et al*. (1996) divided population T and population M into two subspecies (*V*. *ramuliflora ssp*. *Abbreviate* and *V*. *ramuliflora ssp*. *Changbaiensis* (Kitag) and other diploid *V*. *ramuliflora* populations into another subspecies (*V*. *ramuliflora ssp*. *ramuliflora* Maxim Ohwi) based on morphological characters, geographical distribution, chromosome numbers, karyotypes and isoenzyme analyses [7]. Specifically, *V*. *ramuliflora ssp*. *ramuliflora* (Maxim) Ohwi (diploid *V*. *ramuliflora* populations except for population T), with 12 chromosomes, is widely distributed in the Daxinganling, Xiaoxinganling, Wandashan, Zhangguangcailing, Chaibai and Wutai Mountains; by contrast, *V*. *ramuliflora ssp*. *Changbaiensis* (Kitag) (population M), with 24 chromosomes, is endemic to 1500–2000 m of Changbai Mountains; and *V*. *ramuliflora ssp*. *Abbreviate* (population T), with 12 chromosomes, occupies the Liaotung Peninsula. In addition, Li *et al*. (1996) also found that both the Fenghuang and Qian mountain populations of *V*. *ramuliflora ssp*. *abbreviata* on the Liaotung peninsula had similar flower character variations, such as larger flowers (as long as 15 mm), significantly longer back calyx lobes (approximated to the length of calyx tube), condensed anthotaxy (branched or not), and persistent involucres (bigger than stipules). These flower character variations are obviously different from other diploid populations [7].

**Table 1:** Various taxonomic designations of *V*. *ramuliflora* by different author.

Population	Taxonomic designations		
	Fu & Chen (1976)	Xia (1996)	Li *et al*. (1996)
T	*V*. *ramuliflora* f. *abbreviate* (new form)	*V*. *chianshanensis* (new species)	*V*. *ramuliflora ssp*. *Abbreviate* (subspecies)
B	*V*. *ramuliflora*	*V*. *ramuliflora*	*V*. *ramuliflora*
H	*V*. *ramuliflora*	*V*. *ramuliflora*	*V*. *ramuliflora*
M	*V*. *ramuliflora* f. *chianschanensis* (new form)	Form of *Vicia ramuliflora* (new form)	*V*. *ramuliflora ssp*. *Changbaiensis* (Kitag) (subspecies)

T, Qianshan (Liaoning province) population (*V*. *ramuliflora*, 2x); B, Hengdaohezi (Heilongjiang province) population (*V*. *ramuliflora*, 2x); H, Dailing (Heilongjiang province) population (*V*. *ramuliflora*, 2x); M, Changpai Mountains (Jilin province) population (*V*. *ramuliflora*, 4x)

It is well known that in eukaryotes, the ribosomal genes comprise two distinct multigene classes that are composed of tandemly arrayed repeated sequences. The major rDNA (45S) corresponds to the nucleolus organizer region (NOR) and includes the 18S, 5.8S, 28S rRNA gene as well as an intergenic non-transcribed spacer; the minor rDNA is comprised of 5S rRNA gene [11]. Due to the apparent molecular conservation of ribosomal sites among closely related groups of organisms, such sequences can be useful tools for cytotaxonomic and evolutionary studies [12]. During the past few years there has been extensive use and development of fluorescence in situ hybridization (FISH) as a tool for both cytotaxonomic and evolutionary research. The hybridization of specific DNA or RNA sequences in situ to cellular targets attached to microscope slides has allowed for the localization of all major and minor rDNA sites. In addition, random amplified polymorphic DNA (RAPD) has also been used to measure genetic variation for establishing genetic and evolutionary relationships and to generate phylogenetic trees for species, subspecies, and populations [13]. RAPD is generated by applying the polymerase chain reaction (PCR) to genomic DNA samples, using randomly constructed oligonucleotides as primers [14,15].

In the present study, we used FISH to examine the distribution of 5S and 18S rDNA sites in the tetraploid population of *V*. *ramuliflora* and its conspecific diploid counterparts. We also examined differences between the diploid and tetraploid populations at the whole genome level based on RAPD. The combination of RAPD and FISH with 5S and 18S rDNA probes offers a powerful approach to further explore the evolution of *V*. *ramuliflora* at diploid and tetraploid levels. Our specific study aims include: (1) determining the number and location of 18S and 5S rDNA loci in diploids and tetraploids of *V*. *ramuliflora* (2) comparing the distribution of RAPD bands in diploids and tetraploids of *V*. *ramuliflora* (3) identifying the genomic origin of *V*. *ramuliflora* tetraploids, and (4) determining the taxonomic status of the Qian Mountains diploid *V*. *ramuliflora* population.

## Materials and Methods

### Ethics statement

No specific permits were required for the sample collection. The field studies did not involved endangered or protected species.

### Plant materials

**Fig 1:**
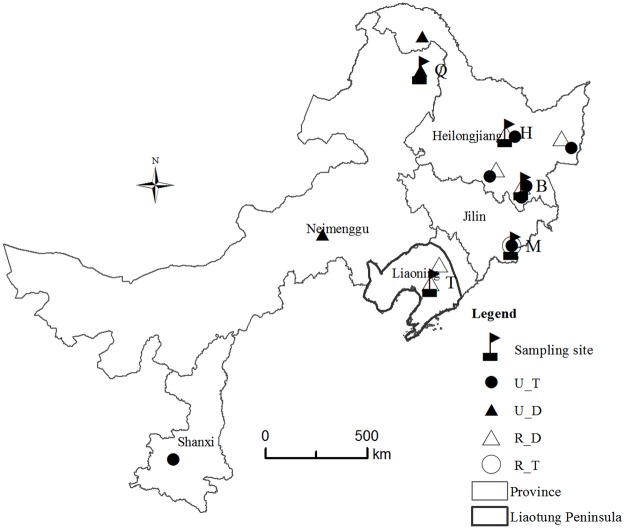
Distribution of *Vicia ramuliflora* and *Vicia unijuga* in China. A white circle represents tetraploid of *V*. *ramuliflora*; A white triangle represents dipoloid of *V*. *ramuliflora*; A black circle represents tetraploid of *V*. *unijuga*; A black triangle represents dipoloid of *V*. *unijuga*; B, Hengdaohezi (Heilongjiang province) population (*V*. *ramuliflora*, 2x); H, Dailing (Heilongjiang province) population (*V*. *ramuliflora*, 2x); T, Qianshan (Liaoning province) population (*V*. *ramuliflora*, 2x); M, Changpai Mountains (Jilin province) population (*V*. *ramuliflora*, 4x); Q, Jiabei (Heilongjiang province) population (*V*. *unijuga*, 2x). Jiabei is located at Neimenggu province, but it belongs to the jurisdiction of Heilongjiang province in administration.

Two species and five populations of Northeast China *Vicia* were analyzed. *Vicia unijuga* was sampled as outgroup. *V*. *unijuga* is closely related species with *V*. *ramuliflora*. Previous studies showed that these two species probably have a common ancestor [7,16]. Diploids of *V*. *unijuga* are distributed closely at Daxinganling in northeast of China. Hence we only selected one *V*. *unijuga* population as outgroup. The identification of *V*. *ramuliflora* and *V*. *unijuga* were based on morphological characters (e.g. their compound leaf structures are dissimilar, with *V*. *unijuga* having only a pair of leaflets and *V*. *ramuliflora* has 2–6 pairs). Detailed information about the materials analyzed is given in Table 2. The mature seeds and young leaves from 13 individuals per population were collected from five native populations in northeast China (Table 2). The distance between populations ranged from 273 km up to 1045 km with specific distances as follows: H and B (273 km) < B and M (298 km) < M and T (437 km) < Q and H (521 km) < H and M (587 km) < B and T (652 km) < Q and B (758 km) < H and T (822 km) < Q and M (974 km) < Q and T (1045 km) (Fig 1). The distance between individuals was kept at least 15 m to increase the possibility of detecting variation among individuals. To determine ploidy, we marked the individuals that we have collected. We collected the leaves and seeds from each marked individual. The ploidy was determined by counting chromosomes at metaphase in root-tip meristem cells taken from germinating seeds.

**Table 2:** Sample sites for 5 populations from *V*. *ramuliflora* and *V*. *Unijuga*.

Population	Locality	No. of plants	Genomic constitution
***V*. *ramuliflora***			
T	Qianshan (Liaoning province)	13	2n = 2x = 12
B	Hengdaohezi (Heilongjiang province)	13	2n = 2x = 12
H	Dailing (Heilongjiang province)	13	2n = 2x = 12
M	Changpai Mountains (Jilin province)	13	2n = 4x = 24
***V*. *unijuga***			
Q	Jiabei (Heilongjiang province)	13	2n = 2x = 12

### Fluorescence in situ hybridization (FISH)

We randomly selected five good metaphases per individual and five individuals per population for constructing an idiogram. We selected three good metaphases from different individual per population for FISH. Root tips were obtained from seeds germinating in Petri dishes. The root tips were pretreated with 0.05% colchicine for 3 h at room temperature. The meristems were fixed in 3:1 ethanol: acetic acid for 24 h at room temperature and stored at -20°C until used for FISH. The FISH procedure was based on Zhang & Sang [17] with minor modifications. Probes used for FISH were 18S rDNA and 5S rDNA from PCR amplification, labeled using a DIG DNA Labeling and Detection Kit (Boehringer, Mannheim, Germany) with biotin-11-dUTP (Sigma) and digoxigenin-11-dUTP (Roche Diagnostics GmbH, Mannheim, Germany), respectively. The biotinylated-probes were detected by avidin-FIFC (Roche Diagnostics GmbH, Mannheim, Germany) and the digoxigenin-labelled probes by anti-digoxigenin rhodamine conjugate (Roche Diagnostics GmbH, Mannheim, Germany). All preparations were counterstained with DAPI (20 μg mL^-1^), mounted in FluoroGuard^™^ antifade reagent and observed with a Leica DMRBE microscope (Leica, Wetzlar, Germany). Fluorescent signals were captured by a SPOT cooled color digital camera system (Diagnostic instruments Inc., MI, USA).

### Random amplified polymorphic DNA (RAPD)

Total DNA was extracted from frozen (-80°C) leaf tissue using the standard CTAB method for small-scale extraction of DNA [15]. Thirteen plants, chosen to represent the entire study material, were initially screened with 80 10-mer RAPD primers from SBS Inc (Beijing China). Seven RAPD primers (Table 3) were selected for further analysis since they yielded polymorphic, clear and reproducible bands. RAPDs were performed in volumes of 10 μl, containing 10 ng of DNA, 1X reaction buffer (TaKaRa, Dalian, China), 1.5 mM MgCl_2_ (TaKaRa Dalian, China), 0.2 μM primer (SBS Beijing, China), 0.2 mM mixture of dNTPs (TaKaRa Dalian, China), and 1.0 U Taq DNA Polymerase (Promega Shanghai, China). The thermocycler (HYBAID) was programmed for 1 cycle of 3 min at 92°C followed by 45 cycles of 1 min at 92°C, 1 min at 40°C, and 2 min at 72°C, and finally by 1 cycle of 10 min at 72°C. The amplified products were separated by electrophoresis in 1.2% agarose gels with a Tris-Boric acid-EDTA (TBE) buffer system. Gels were stained with ethidium bromide and photographed on AmpGene imaging devices for further analyses. Molecular Weight Marker DL2000 (TaKaRa Dalian, China) was used to determine the size of the DNA fragments. To prevent other DNA contamination, negative controls (i.e. the reaction mixture without genomic DNA) were run in each PCR run. To ensure reproducibility between runs, DNA from the same three plants was included in every PCR run. DNA from three additional plants was amplified twice in each PCR run as a control of reproducibility within runs.

**Table 3:** List of RAPD primers used and their nucleotide sequences.

No. of primers	Sequence (5’—3’)
**Used for RAPDs**	
SBSA10	GTGATCGCAG
SBSP10	TCCCGCCTAC
SBSP17	TGACCCGCCT
SBSQ18	AGGCTGGGTG
SBSQ4	AGTGCGCTGA
SBSQ5	CCGCGTCTTG
SBSQ15	GGGTAACGTG

### Data analysis

#### RAPD data analysis

Electrophoretic data were scored manually, and each band in the RAPD profile was treated as an independent character (locus) with two states (alleles), presence (1) or absence (0). Finally, a binary data matrix (S1 Table) was constructed to be used for statistical analysis. All RAPD data analyses (Dice’s coefficient for constructing UPGMA and PCO of individual level, percentage of polymorphic bands, pairwise PHiST coefficients for constructing UPGMA of population level) were based on this binary data matrix. Among the various similarity indices, those of Jaccard and Dice were chosen as most appropriate for dominant markers since they do not attribute any genetic meaning to the coincidence of band absence. Dice’s coefficient of similarity was calculated using the NTsys-pc (NTsys-pc software version 2.10) [18]. These matrices of Dice’s coefficient were then used to perform a UPGMA cluster analysis and principal coordinates (PCO) analysis of individual level (NTsys-pc software version 2.10) [18]. The percentage of polymorphic bands (PPB) was calculated by the PopGene program [19]. Pairwise PhiST coefficients, which are interpreted as analogous to Fst values, were computed at the population level (WinAmova version 1.55) [20]. Bootstrapping was carried out using the bootstrap function in PowerMarker [21] and consensus trees were created using the consense program in the PHYLIP software package v3.695 [22]. Two methods, UPGMA and Neighbor-Joining, were used to create the dendrograms.

## Results

### FISH with 18S and 5S ribosomal DNA probes

**Fig 2:**
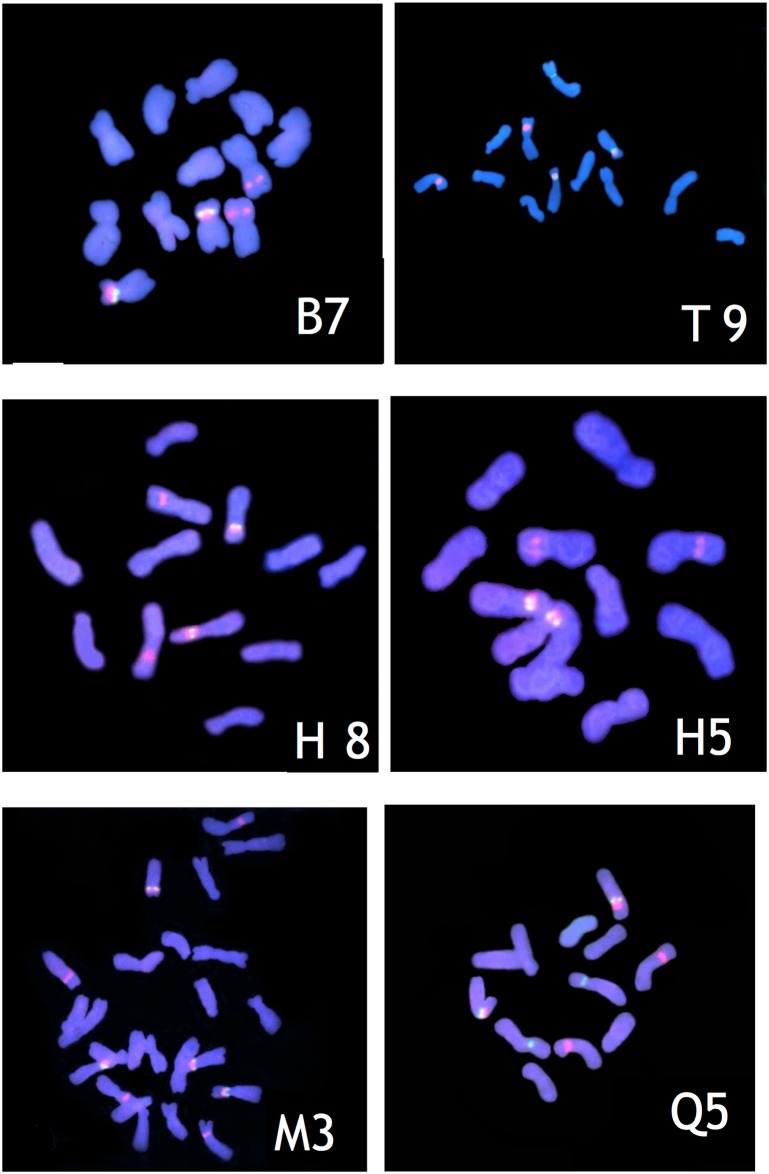
FISH of 18S rDNA (red) and 5S rDNA (green) to somatic C-metaphase chromosomes of 5 populations. B, Hengdaohezi (Heilongjiang province) population (*V*. *ramuliflora*, 2x); T, Qianshan (Liaoning province) population (*V*. *ramuliflora*, 2x); H, Dailing (Heilongjiang province) population (*V*. *ramuliflora*, 2x); M, Changpai Mountains (Jilin province) population (*V*. *ramuliflora*, 4x); Q, Jiabei (Heilongjiang province) population (*V*. *unijuga*, 2x). Scale bar = 10 μm.

**Fig 3:**
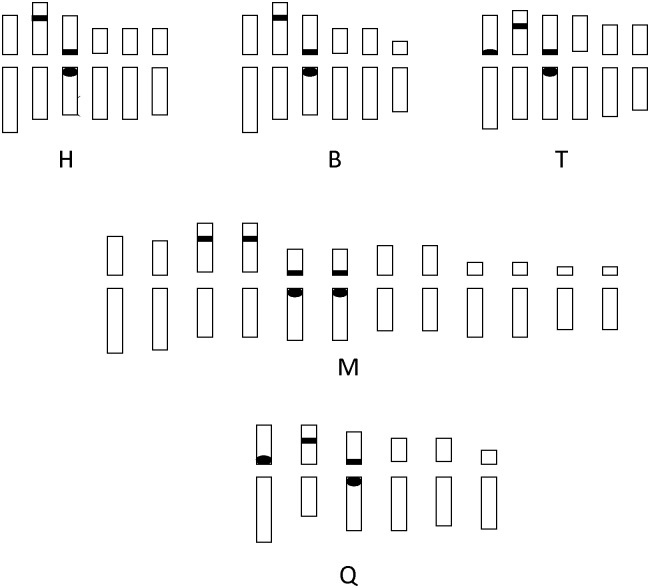
Idiograms of 5 *Vicia* populations. H, Dailing (Heilongjiang province) population (*V*. *ramuliflora*, 2x); B, Hengdaohezi (Heilongjiang province) population (*V*. *ramuliflora*, 2x); T, Qianshan (Liaoning province) population (*V*. *ramuliflora*, 2x); M, Changpai Mountains (Jilin province) population (*V*. *ramuliflora*, 4x); Q, Jiabei (Heilongjiang province) population (*V*. *unijuga*, 2x). A black rectangle represents 18s rDNA present in two of homologous chromosomes; A black circle represents 5s rDNA present in two of homologous chromosomes; A black oval represents 5s rDNA present in one of homologous chromosomes;

18S rDNA in-situ hybridizations revealed similar signals among five diploid *V*. *ramuliflora* populations (Figs 2 and 3). In the five diploids examined, the number and location of 18S rDNA was identical: One pair of 18S rDNA signals was located at the secondary constriction of the short arm of chromosome 2; another pair of 18S rDNA signals was distributed at the centromere zone of chromosome 3. By contrast, in the tetraploid population (M), two pairs of 18S rDNA signals were located at the secondary constriction of the short arm of chromosomes 3 and 4, and another two pairs of 18S rDNA signals were located at the centromere zone of chromosomes 5 and 6. The number of 18S rDNA signals in the tetraploid population was twice that in each diploid population. However, the position of 18S rDNA signals was identical in tetraploid and diploid populations.

The number and location of 5S rDNA in population B and H was identical: Only one pair of 5S rDNA signals was located at the centromere zone of chromosome 3. However, the number and location of 5S rDNA in population T and Q was different from population B and H: Besides one pair of 5S rDNA signals was located at the centromere zone of chromosome 3, a single 5S rDNA signal in population T and one pair of 5S rDNA signals in population Q were also been found at the centromere zone of chromosome 1. By contrast, in the tetraploid population (M), two pairs of 5S rDNA signals were located at the centromere zone of chromosomes 5 and 6. The number of 5S rDNA signals in the tetraploid population was twice that in diploid population B or diploid population H. However, the position of 5S rDNA signals was identical in the tetraploid population and diploid populations B and H. For all populations, the linkage pattern of these two types of rDNAs is: 5S rDNA is adjacent with 18S rDNA on the same arm of the same chromosome. 18S rDNA is near outside.

### RAPD analysis with seven primers

#### The patterns of amplification bands

The total number of reproducible bands amplified with seven primers was 109, with a size range from 100 to 2000 bp and quantity range from 8 to 21 per primer. The number of polymorphic loci and the percentage of polymorphic loci of each population are presented in Table 4.

**Table 4:** The number of polymorphic loci and the percentage of polymorphic loci of each population.

Population	The number of polymorphic loci	The percentage of polymorphic loci
T	33	30.28%
B	57	52.29%
H	72	66.06%
M	33	30.28%
Q	53	48.62%

T, Qianshan (Liaoning province) population (*V*. *ramuliflora*, 2x); B, Hengdaohezi (Heilongjiang province) population (*V*. *ramuliflora*, 2x); H, Dailing (Heilongjiang province) population (*V*. *ramuliflora*, 2x); M, Changpai Mountains (Jilin province) population (*V*. *ramuliflora*, 4x); Q, Jiabei (Heilongjiang province) population (*V*. *unijuga*, 2x)

The occurrence ratio of amplification band at per locus in 13 individuals of each population is presented in Table 5. Diploid population H owned 16 specific bands, with the ratio of the individuals with specific bands at per specific locus ranging from 46% to 8%. In population H, the occurrence ratio of amplification band at three loci (A10-2, A10-8 and Q5-11) was 0%, indicating that population H has evolved distinct DNA sequences compared to other populations examined. Diploid population B owned seven specific bands, with the ratio of the individuals with specific bands at per specific locus ranging from 23% to 8%. Comparing across populations, only in population B was the occurrence ratio of amplification bands at three loci (A10-4, P17-2 and P17-6) 0%. Diploid population T owned four specific bands, with the ratio of the individuals with specific bands at per specific locus ranging from 54% to 8%. Compared to other populations, only in population T was the occurrence ratio of amplification band at four loci (P10-14, Q18-3, Q5-5 and Q15-11) 0%. Diploid population Q owned 10 specific bands, with the ratio of the individuals with specific bands at per specific locus ranging from 92% to 8%. The occurrence ratio of amplification bands at four loci (Q4-9, Q5-8, Q5-16 and Q15-8) was 0% in population Q. The tetraploid population M owned only one specific band, with the ratio of the individuals with a specific band at this specific locus being 46%. Interestingly, the occurrence ratio of amplification bands at two loci (P17-7 and Q15-12) was 0% only in tetraploid population M, indicating that tetraploid population M has evolved distinct DNA sequences compared to other diploid populations. While both the tetraploid population (M) and four diploid populations (B, H, T and Q) have evolved distinct DNA sequences, the five populations all owned bands at A10-1, A10-6, A10-9, P10-2, P10-8, P10-17, P17-9, P17-14, Q18-2 and Q18-8 loci, although the occurrence ratio of amplification bands at these loci in each individual of each population was different. These patterns indicated that the DNA sequences were high conserved at these loci and showed common genus characters.

**Table 5:** The occurrence ratio of amplification band at per locus in 13 individuals of each population.

RAPD Locus	Population				
	M	H	B	T	Q
A10-1	8%	23%	31%	8%	8%
A10-2	23%	0%	8%	31%	31%
A10-3	8%	8%	8%	0%	0%
A10-4	15%	31%	0%	38%	31%
A10-5	0%	0%	15%	0%	0%
A10-6	92%	85%	100%	92%	85%
A10-7	0%	15%	0%	0%	23%
A10-8	15%	0%	8%	8%	46%
A10-9	23%	77%	54%	54%	15%
A10-10	0%	31%	23%	0%	0%
A10-11	0%	0%	0%	23%	0%
A10-12	8%	8%	0%	0%	15%
A10-13	0%	15%	8%	15%	0%
A10-14	0%	0%	8%	0%	15%
A10-15	0%	8%	0%	0%	0%
A10-16	0%	0%	0%	8%	0%
A10-17	0%	0%	0%	0%	15%
A10-18	0%	8%	0%	0%	0%
P10-1	8%	23%	15%	0%	0%
P10-2	31%	62%	77%	15%	100%
P10-3	0%	0%	8%	0%	0%
P10-4	0%	15%	0%	0%	0%
P10-5	15%	15%	0%	0%	8%
P10-6	0%	0%	31%	0%	8%
P10-7	0%	8%	0%	0%	0%
P10-8	54%	8%	31%	15%	15%
P10-9	0%	31%	8%	0%	0%
P10-10	0%	8%	31%	0%	0%
P10-11	0%	31%	0%	8%	15%
P10-12	0%	38%	0%	0%	85%
P10-13	0%	15%	0%	46%	31%
P10-14	15%	46%	62%	0%	100%
P10-15	38%	0%	0%	0%	8%
P10-16	0%	0%	8%	0%	0%
P10-17	100%	100%	92%	100%	8%
P17-1	23%	23%	0%	0%	15%
P17-2	31%	31%	0%	8%	69%
P17-3	0%	15%	0%	0%	77%
P17-4	23%	0%	0%	62%	15%
P17-5	0%	8%	8%	0%	0%
P17-6	100%	46%	0%	62%	85%
P17-7	0%	77%	100%	92%	31%
P17-8	0%	8%	8%	0%	46%
P17-9	100%	69%	38%	8%	8%
P17-10	0%	15%	0%	0%	0%
P17-11	0%	15%	0%	0%	0%
P17-12	46%	0%	0%	0%	0%
P17-13	0%	8%	0%	0%	0%
P17-14	54%	92%	31%	77%	46%
Q18-1	0%	0%	38%	0%	85%
Q18-2	92%	69%	77%	100%	100%
Q18-3	15%	54%	8%	0%	15%
Q18-4	0%	0%	0%	0%	31%
Q18-5	0%	0%	15%	0%	54%
Q18-6	0%	69%	38%	0%	85%
Q18-7	0%	0%	0%	0%	23%
Q18-8	8%	46%	54%	92%	8%
Q4-1	0%	0%	0%	0%	8%
Q4-2	38%	38%	15%	0%	0%
Q4-3	0%	0%	0%	8%	69%
Q4-4	0%	0%	0%	0%	8%
Q4-5	0%	23%	23%	0%	0%
Q4-6	0%	0%	0%	0%	8%
Q4-7	0%	69%	8%	0%	0%
Q4-8	0%	0%	0%	0%	15%
Q4-9	38%	23%	15%	38%	0%
Q4-10	0%	31%	8%	0%	0%
Q4-11	0%	46%	0%	0%	0%
Q4-12	0%	54%	15%	15%	0%
Q4-13	0%	0%	15%	0%	0%
Q4-14	15%	62%	15%	0%	0%
Q4-15	0%	0%	15%	0%	0%
Q4-16	0%	0%	0%	31%	0%
Q4-17	0%	8%	0%	0%	0%
Q4-18	8%	8%	0%	0%	8%
Q4-19	0%	0%	0%	0%	92%
Q4-20	0%	8%	0%	0%	0%
Q4-21	0%	8%	0%	0%	0%
Q5-1	0%	0%	8%	0%	0%
Q5-2	0%	0%	23%	38%	0%
Q5-3	0%	15%	0%	0%	0%
Q5-4	0%	38%	0%	0%	0%
Q5-5	8%	77%	85%	0%	31%
Q5-6	0%	0%	0%	54%	0%
Q5-7	0%	0%	0%	0%	8%
Q5-8	92%	31%	46%	23%	0%
Q5-9	0%	8%	0%	0%	0%
Q5-10	0%	31%	15%	0%	0%
Q5-11	8%	0%	8%	46%	77%
Q5-12	15%	15%	8%	0%	0%
Q5-13	0%	23%	8%	0%	0%
Q5-14	0%	0%	23%	0%	0%
Q5-15	0%	8%	8%	15%	0%
Q5-16	8%	92%	85%	85%	0%
Q5-17	0%	38%	38%	46%	0%
Q15-1	0%	15%	8%	0%	0%
Q15-2	8%	0%	0%	0%	8%
Q15-3	0%	15%	23%	0%	77%
Q15-4	0%	0%	0%	0%	62%
Q15-5	0%	0%	23%	0%	8%
Q15-6	0%	15%	0%	0%	0%
Q15-7	0%	8%	0%	0%	8%
Q15-8	100%	62%	85%	92%	0%
Q15-9	0%	8%	0%	0%	0%
Q15-10	0%	15%	0%	0%	62%
Q15-11	8%	38%	8%	0%	38%
Q15-12	0%	8%	15%	8%	8%
Q15-13	0%	0%	8%	0%	85%
Q15-14	0%	15%	0%	0%	0%

T, Qianshan (Liaoning province) population (*V*. *ramuliflora*, 2x); B, Hengdaohezi (Heilongjiang province) population (*V*. *ramuliflora*, 2x); H, Dailing (Heilongjiang province) population (*V*. *ramuliflora*, 2x); M, Changpai Mountains (Jilin province) population (*V*. *ramuliflora*, 4x); Q, Jiabei (Heilongjiang province) population (*V*. *unijuga*, 2x

#### Genetic distances and principal coordinates analysis

**Fig 4:**
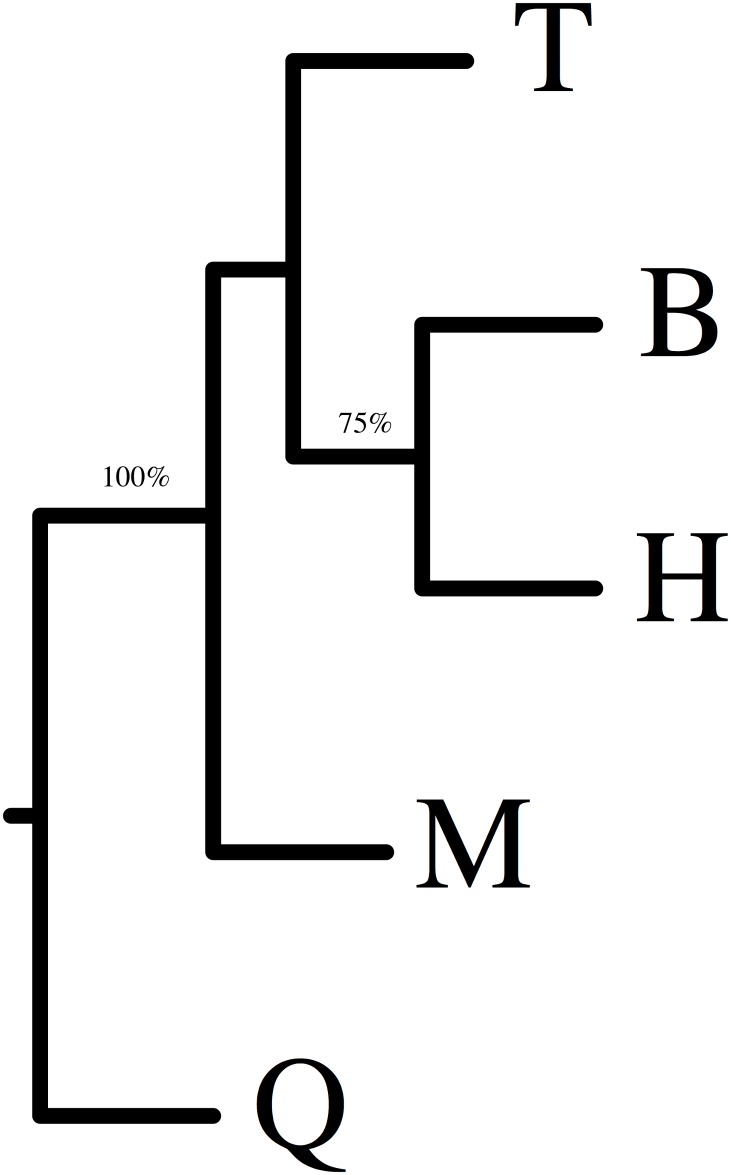
Dendrogram of five populations from *V*. *ramuliflora* and *V*. *unijuga* based on RAPD markers with UPGMA analysis. B, Hengdaohezi (Heilongjiang province) population (*V*. *ramuliflora*, 2x); H, Dailing (Heilongjiang province) population (*V*. *ramuliflora*, 2x); T, Qianshan (Liaoning province) population (*V*. *ramuliflora*, 2x); M, Changpai Mountains (Jilin province) population (*V*. *ramuliflora*, 4x); Q, Jiabei (Heilongjiang province) population (*V*. *unijuga*, 2x). The branches with bootstrap values of greater than 50% are marked. The numbers at the nodes indicate the percentage number of 1000 bootstrap replications.

**Fig 5:**
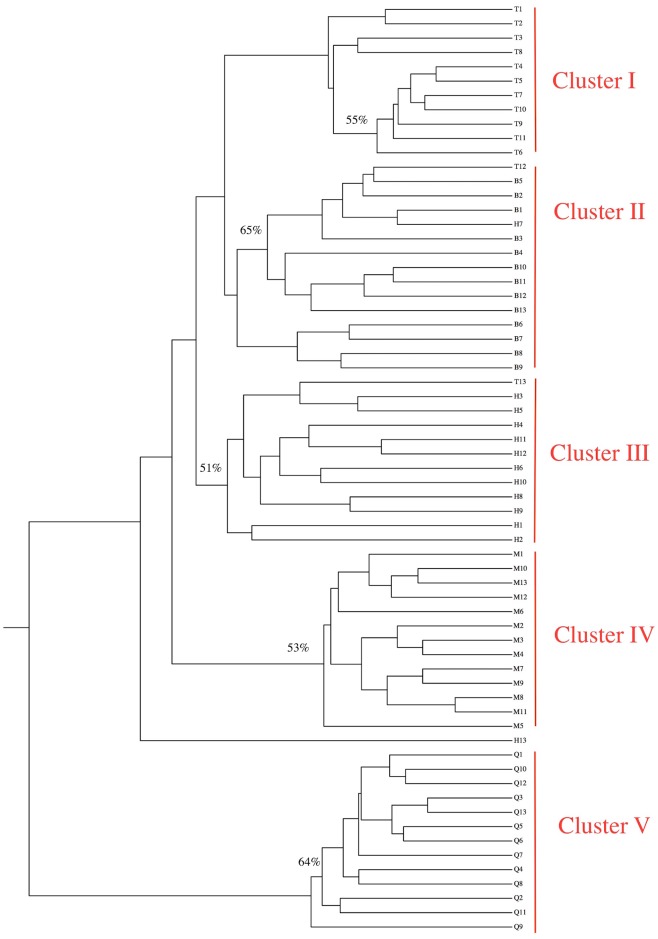
Dendrogram showing the relationships among all plants from five populations of *V*. *ramuliflora* and *V*. *unijuga* based on RAPD markers with UPGMA method. B, Hengdaohezi (Heilongjiang province) population (*V*. *ramuliflora*, 2x); H, Dailing (Heilongjiang province) population (*V*. *ramuliflora*, 2x); T, Qianshan (Liaoning province) population (*V*. *ramuliflora*, 2x); M, Changpai Mountains (Jilin province) population (*V*. *ramuliflora*, 4x); Q, Jiabei (Heilongjiang province) population (*V*. *unijuga*, 2x). The branches with bootstrap values of greater than 50% are marked. The numbers at the nodes indicate the percentage number of 1000 bootstrap replications.

The genetic distance of each two diploid populations was: B and H < B and T < T and H < H and Q < B and Q < T and Q (Table 6). The genetic distances between the tetraploid population and each of the diploid populations was: M and H < M and B < M and T < M and Q. A dendrogram constructed by UPGMA method, based on genetic distances (PhiST), indicated that population B and H showed the closest relationships, tetraploid population M grouped with population B, H, and T and population Q was most distantly related to other populations (Fig 4). By contrast, a dendrogram constructed by Neighbor-Joining indicated that population B and H group together and population M and T group together (S1 Fig). In addition, a dendrogram constructed for 65 individuals, using Dice’s coefficient of similarity and UPGMA clustering, showed individuals dividing into two main groups: namely all 52 individuals from *V*. *ramuliflora* formed one major group, while thirteen individuals from *V*. *unijuga* formed another distinct group (Fig 5). Furthermore, the *V*. *ramuliflora* group was further subdivided into four clusters: Cluster I contained eleven individuals from population T (T12 and T13 were beyond of Cluster I); Cluster II contained all thirteen individuals from population B, T12, and H7; Cluster III contained eleven individuals from population H (H7 and H13 were beyond of Cluster III) and T13; Cluster IV contained all thirteen individuals from tetraploid population M. By contrast, a dendrogram constructed for 65 individuals (S2 Fig), using Neighbor-Joining, showed that all individuals formed four clusters: Cluster A contained all thirteen individuals from population M; Cluster B contained all thirteen individuals from population T; Cluster C contained twenty three individuals from population B and H (H13, H4 and B3 were beyond of Cluster C); Cluster D contained all thirteen individuals from population Q.

**Table 6:** PhiST genetic distances of five populations.

**Population**	**T**	**B**	**H**	**M**	**Q**
**T**	0.0000				
**B**	0.2230	0.0000			
**H**	0.2470	0.1136	0.0000		
**M**	0.3446	0.2991	0.2739	0.0000	
**Q**	0.4993	0.3993	0.3701	0.4925	0.0000

T, Qianshan (Liaoning province) population (*V*. *ramuliflora*, 2x); B, Hengdaohezi (Heilongjiang province) population (*V*. *ramuliflora*, 2x); H, Dailing (Heilongjiang province) population (*V*. *ramuliflora*, 2x); M, Changpai Mountains (Jilin province) population (*V*. *ramuliflora*, 4x); Q, Jiabei (Heilongjiang province) population (*V*. *unijuga*, 2x)

**Fig 6:**
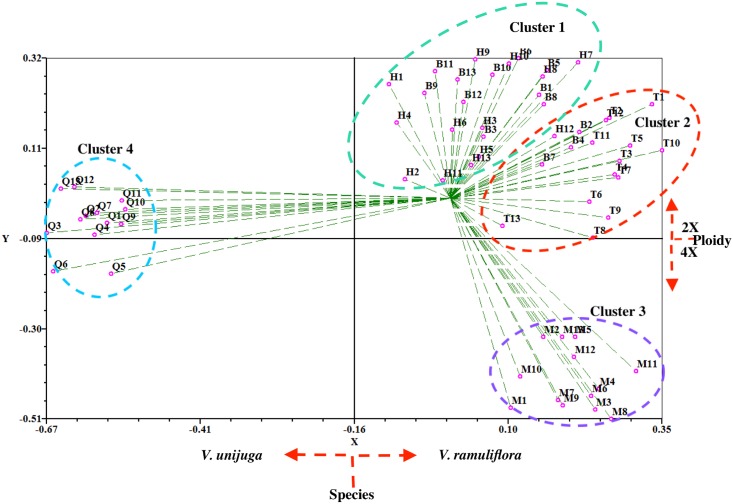
Two-dimensional plot from a PCO based on DICE cofficients among plants of *V*. *ramuliflora* and *V*. *unijuga* by RAPD markers. B, Hengdaohezi (Heilongjiang province) population (*V*. *ramuliflora*, 2x); H, Dailing (Heilongjiang province) population (*V*. *ramuliflora*, 2x); T, Qianshan (Liaoning province) population (*V*. *ramuliflora*, 2x); M, Changpai Mountains (Jilin province) population (*V*. *ramuliflora*, 4x); Q, Jiabei (Heilongjiang province) population (*V*. *unijuga*, 2x)

The PCO scatter plot (Fig 6) assigned all 65 individuals to four major clusters: Cluster 1 included most individuals from population B and H; Cluster 2 was composed of all 13 individuals from population T, 3 individuals from population B, and one individual from population H; Cluster 3 consisted of all 13 individuals from tetraploid population M; Cluster 4 comprised all 13 individuals from population Q. Like the UPGMA clustering, the first principal coordinate axis (X axis) of the PCO plot showed key variation between individuals into *V*. *ramuliflora* group and *V*. *unijuga* group. The second principal coordinate axis (Y axis) further separated all individuals into diploid and tetraploid groups.

## Discussion

### The evolution of *V*. *ramuliflora* at tetraploid level

Revealing the origins of polyploidy is quite difficult because the genome often rapidly changed by chromosomal rearrangements, diploidization, and inter-genomic invasion after polyploidization [23–27]. Our FISH-based study showed that the number of 18S and 5S rDNA hybridization signal in the tetraploid population (M) of *V*. *ramuliflora* was double that in the diploid populations B or H. However, the hybridization patterns of the18S and 5S rDNA probes in diploid population B, diploid population H, and tetraploid population M were identical: all hybridization signals were all located at the same regions of corresponding chromosomes (Figs 2 and 3). By contrast, diploid population T had an additional single 5S rDNA signal at the centromere zone of chromosome 1. Thus, our FISH results imply that population B and H are more likely the parents of tetraploid M. However, our cluster analysis (Figs 4 and 5, S1 and S2 Figs) and PCO analysis based on RAPD (Fig 6) showed that the tetraploid population M had more close relationships with diploid population T. This difference between FISH and RAPD probably was caused by chromosomal rearrangements, diploidization, and inter-genomic invasion after polyploidization [23–27]. Thus, genomic origin of tetraploid population M is still ambiguous and in need of further study. Previous flower biology and breeding system research [28, 29] has indicated that *Vicia* species in Sect *Vicilla* are self-pollinated plants. Taken together, we think that the tetraploid *V*. *ramuliflora* is an autotetraploid probably derived by the union of unreduced gametes. What may have driven the tetraploid status in *V*. *ramuliflora*? Studies showed that the frequency of 2n gametes is subject to biological characteristics of the plant and genetic factors, but also external environmental factors, such as temperature, water and nutrients [30,31,32]. For example, Belling (1925) found the frequency of 2n gametes sharply increased in plants of *Datura strastramonium*, *Uvularia grandiflora*, and *strizolobium* during cold spells [30]. Similarly, Grant (1952) discovered that the frequency of 2n gametes of the plants of *Gilia* in arid soils was as much as nine hundred times of that in fertile soils [32]. Collectively, these studies indicate that environmental selection pressure is an important factor for the formation of 2n gametes, which may be why many polyploid species are distributed at high latitude and high altitude zones or other harsh environments. In China, tetraploid of *V*. *ramuliflora* are limited only being present above 2000 m mountains, which have extreme climatic conditions. In addition, mountain glaciers occurred many times at some high mountains in Asia in the Quaternary Period, which together with extreme climatic conditions may have promoted the formation of 2n gametes. Previous many studies found that polyploids are often better adapted to harsher environmental conditions [33–36]. For example, Kay (1969) found that polyploids are often better adapted to dry conditions [33] and Liu et al. (2011) found that polyploids show increased cold tolerance [35]. Thus, we suspect that the tetraploid M population is better adapted to the harsher, high mountain environmental conditions compared with diploid populations which occur at lower elevations.

### The evolution of *V*. *ramuliflora* at diploid level

Previous researchers have had different opinions on the taxonomic status of diploid *V*. *ramuliflora* populations [9,10,7]. Fu & Chen (1976) thought that the Qianshan diploid population (T) and the Changbaishan tetraploid population (M) should be divided as different forms of *V*. *ramuliflora* [9]. Xia (1996) thought that the diploid T population should be treated as an independent species, while the tetraploid M population should be treated as a form of *V*. *ramuliflora* [10]. Li (1996) suggested that the diploid T population and the tetraploid M population should be divided as two diffferent subspecies of *V*. *ramuliflora* [7]. Our FISH study showed that 18S rDNA had same hybridization pattern and hybridization numbers for all diploid populations. However, 5s rDNA showed variety among diploid populations: population B and population H only have a pair of 5s rDNA signals at centromere of the third pair of chromosomes. By contrast, population T and population Q have additional 5s rDNA signals at centromere of the first pair of chromosomes besides 5s rDNA signals at centromere of the third pair of chromosomes (Figs 2 and 3). Cluster analysis and PCO analysis based on RAPD also showed that population T are different from population B and population H (Figs 4, 5 and 6), although T12 and T13 clustered with population B and population H based on UPGMA method (Fig 5). To further our inference, we also used Neighbor-Joining method to cluster at the population and individual level. Neighbor-Joining based clustering showed that all individuals from population T grouped together and formed an independent cluster (cluster B) (most bootstrap values are very low for clustering at individual level, reflecting high genetic diversity among individuals) (S1 and S2 Figs). This indicated that the diploid population T was different from other diploid populations (B and H). These molecular-based results are also consistent with previous studies based on morphological traits [10,7]. On the other hand, although population T was different with other diploid populations, it still clustered within *V*. *ramuliflora* species. Thus, we do not support its elevation as a new species. Instead, we support the taxon treatment of Li (1996) and suggest that the Qianshan population (T) be considered as a new subspecies. It is important to note that the location of the 5S rDNA in population T (*V*. *ramuliflora*) is similar to that of population Q (*V*. *unijuga*). Previous studies have also found that population T of *V*. *ramuliflora* and *V*. *unijuga* have similar morphological characters. Usually, *V*. *ramuliflora* and *V*. *unijuga* have different compound leaf structures: *V*. *unijuga* has only a single pair of leaflets and *V*. *ramuliflora* has two to six pairs. However, Li et al. (1996) found that some exceptional individuals of *V*. *unijuga* posess three leaflets and co-occur with normal *V*. *unijuga* individuals in both the Tayuan and Maorshan populations. They also observed a change in the compound leaf structure during the growth of seedlings and found that the compound leaf consists of a pair of leaflets for 1-year-old seedlings in both *V*. *unijuga* and *V*. *ramuliflora*. Based on 5S rDNA locations and compound leaf structure, we presume that these two species likely have a common ancestor.

## Conclusions

Our FISH-based study showed that the tetraploid cytotype of *V*. *ramuliflora* at Changbai Mountains (M) had identical 18S and 5S rDNA distribution pattern with the diploid Hengdaohezi population (B) and the diploid Dailing population (H). However, UPGMA clustering, Neighbor-Joining clustering, principal coordinates analysis based on RAPD showed that the tetraploid cytotype (M) had close relationships with diploid population T. Based on our study results and the fact that interspecific hybridization among *Vicia* species is known to be limited, we think that the tetraploid *V*. *ramuliflora* is autotetraploid, but its likely parents still need to be studied further. In addition, our study also found that Qianshan diploid population (T) had evolved distinct 5s rDNA sites compared with other diploid populations. Specific band pattern, genetic distances, UPGMA clustering, principal coordinates analysis based on RAPD also separated Qianshan diploid population (T) from other diploid populations (B and H). We, therefore, suggest that diploid population T be re-classified as a distinct subspecies.

## Supporting Information

S1 Table Binary data matrix based on RAPD. Electrophoretic data were scored manually, and each band in the RAPD profile was treated as an independent character (locus) with two states (alleles), presence (1) or absence (0). B, Hengdaohezi (Heilongjiang province) population (*V*. *ramuliflora*, 2x); H, Dailing (Heilongjiang province) population (*V*. *ramuliflora*, 2x); T, Qianshan (Liaoning province) population (*V*. *ramuliflora*, 2x); M, Changpai Mountains (Jilin province) population (*V*. *ramuliflora*, 4x); Q, Jiabei (Heilongjiang province) population (*V*. *unijuga*, 2x). SBSA10, SBSP10, SBSP17, SBSQ18, SBSQ4, SBSQ5, SBSQ15 are the primers used for RAPDs. Their sequences were listed in Table 3.
(XLS)

S2 Table Structural and morphological characters of chromosomes of five populations from *V*. *ramuliflora* and *V*. *unijuga*. B, Hengdaohezi (Heilongjiang province) population (*V*. *ramuliflora*, 2x); H, Dailing (Heilongjiang province) population (*V*. *ramuliflora*, 2x); T, Qianshan (Liaoning province) population (*V*. *ramuliflora*, 2x); M, Changpai Mountains (Jilin province) population (*V*. *ramuliflora*, 4x); Q, Jiabei (Heilongjiang province) population (*V*. *unijuga*, 2x). RL, Relative length; RLA, Relative long arm; RSA, Relative short arm; AR, Arm ratio; CP, Centromeric position; m, metacentrics; st, subtelocentrics; sm, submetacentrics.
(DOC)

S1 Fig Dendrogram of five populations from *V*. *ramuliflora* and *V*. *unijuga* based on RAPD markers with Neighbor-Joining analysis. B, Hengdaohezi (Heilongjiang province) population (*V*. *ramuliflora*, 2x); H, Dailing (Heilongjiang province) population (*V*. *ramuliflora*, 2x); T, Qianshan (Liaoning province) population (*V*. *ramuliflora*, 2x); M, Changpai Mountains (Jilin province) population (*V*. *ramuliflora*, 4x); Q, Jiabei (Heilongjiang province) population (*V*. *unijuga*, 2x). The branches with bootstrap values of greater than 50% are marked. The numbers at the nodes indicate the percentage number of 1000 bootstrap replications.
(JPG)

S2 Fig Dendrogram showing the relationships among all plants from five populations of *V*. *ramuliflora* and *V*. *unijuga* based on RAPD markers with Neighbor-Joining method. B, Hengdaohezi (Heilongjiang province) population (*V*. *ramuliflora*, 2x); H, Dailing (Heilongjiang province) population (*V*. *ramuliflora*, 2x); T, Qianshan (Liaoning province) population (*V*. *ramuliflora*, 2x); M, Changpai Mountains (Jilin province) population (*V*. *ramuliflora*, 4x); Q, Jiabei (Heilongjiang province) population (*V*. *unijuga*, 2x). The branches with bootstrap values of greater than 50% are marked. The numbers at the nodes indicate the percentage number of 1000 bootstrap replications.
(JPG)
