# Supplementary material for: The Evolution of Vicia ramuliflora (Fabaceae) at Tetraploid and Diploid Levels Revealed with FISH and RAPD
Source: PLoS One. 2017 Jan 30;12(1):e0170695. doi: 10.1371/journal.pone.0170695 (PMC5279728; doi:10.1371/journal.pone.0170695)
Supplement: S2 Table — B, Hengdaohezi (Heilongjiang province) population (V. ramuliflora, 2x); H, Dailing (Heilongjiang province) population (V. ramuliflora, 2x); T, Qianshan (Liaoning province) population (V. ramuliflora, 2x); M, Changpai Mountains (Jilin province) population (V. ramuliflora, 4x); Q, Jiabei (Heilongjiang province) population (V. unijuga, 2x). RL, Relative length; RLA, Relative long arm; RSA, Relative short arm; AR, Arm ratio; CP, Centromeric position; m, metacentrics; st, subtelocentrics; sm, submetacentrics. (DOC) [file pone.0170695.s002.doc]

**S2 Table**

| Population | Chromosome | RLA+RSA= RL | AR | CP |
| --- | --- | --- | --- | --- |
| H | 1  2  3  4  5  6 | 14.44+7.70=22.14  10.16+9.52=19.68  9.86+5.76=15.62  10.26+4.68=14.94  10.18+4.06=14.24  8.60+4.30=12.90 | 1.86  1.07  1.71  2.19  2.51  2.01 | sm  m  sm  sm  sm  sm |
| B  p | 1  2  3  4  5  6 | 14.53+7.20=21.73  9.18+8.87=18.05  10.80+4.52=15.40  10.85+4.22=15.07  10.37+4.07=14.44  10.03+2.82=12.85 | 2.02  1.03  2.41  2.57  2.55  3.56 | sm  m  sm  sm  sm  st |
| T  pop | 1  2  3  4  5  6 | 13.38+6.82=20.20  10.24+7.94=18.18  10.30+6.54=16.84  10.10+6.00=16.10  10.05+5.03=17.08  10.26+4.30=14.56 | 1.96  1.29  1.57  1.68  2.00  2.39 | sm  m  m  m  sm  sm |
| M | 1  2  3  4  5  6  7  8  9  10  11  12 | 6.70+3.62=10.32  6.61+3.38=9.99  4.90+4.69=9.59  4.84+4.53=9.40  5.87+2.20=8.85  5.65+2.20=8.85  5.50+2.28=7.78  5.54+2.09=7.63  6.22+1.37=7.59  6.21+1.24=7.45  6.20+1.12=7.32  5.99+1.03=7.02 | 1.80  1.96  1.04  1.08  2.57  2.57  2.41  2.65  4.54  5.01  5.54  5.82 | sm  sm  m  m  sm  sm  sm  sm  st  st  st  st |
| Q | 1  2  3  4  5  6 | 13.99+6.79=20.78  9.65+8.47=18.12  10.47+5.30=15.77  10.47+5.01=15.48  10.58+4.57=15.15  11.92+2.78=14.70 | 2.06  1.14  1.97  2.09  2.32  4.28 | sm  m  sm  sm  sm  st |
