# Supplementary figures and images for: The Evolution of Vicia ramuliflora (Fabaceae) at Tetraploid and Diploid Levels Revealed with FISH and RAPD
Source: PLoS One. 2017 Jan 30;12(1):e0170695. doi: 10.1371/journal.pone.0170695 (PMC5279728; doi:10.1371/journal.pone.0170695)

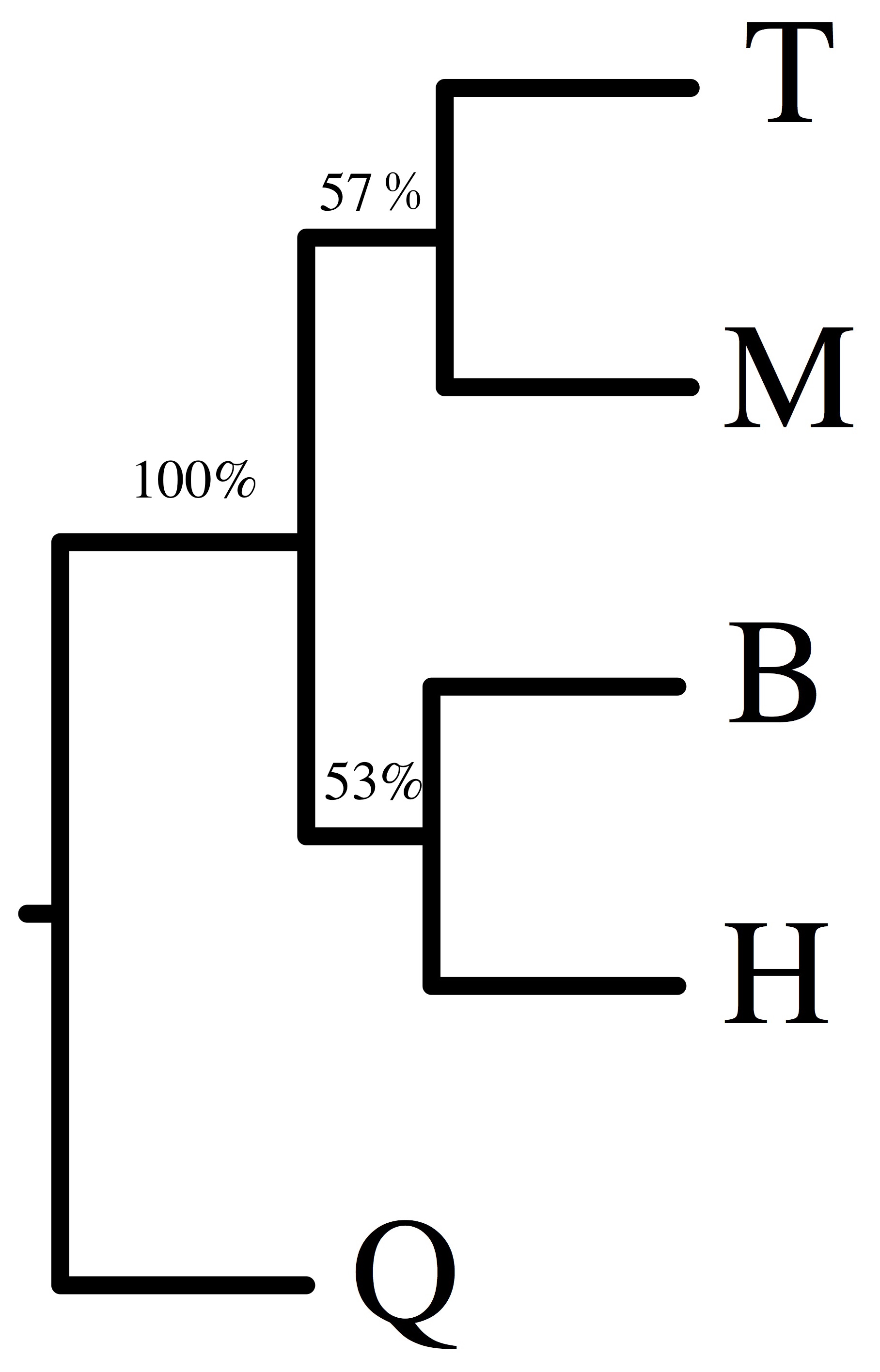

Supplement: S1 Fig — B, Hengdaohezi (Heilongjiang province) population (V. ramuliflora, 2x); H, Dailing (Heilongjiang province) population (V. ramuliflora, 2x); T, Qianshan (Liaoning province) population (V. ramuliflora, 2x); M, Changpai Mountains (Jilin province) population (V. ramuliflora, 4x); Q, Jiabei (Heilongjiang province) population (V. unijuga, 2x). The branches with bootstrap values of greater than 50% are marked. The numbers at the nodes indicate the percentage number of 1000 bootstrap replications. (JPG) [file pone.0170695.s003.jpg]

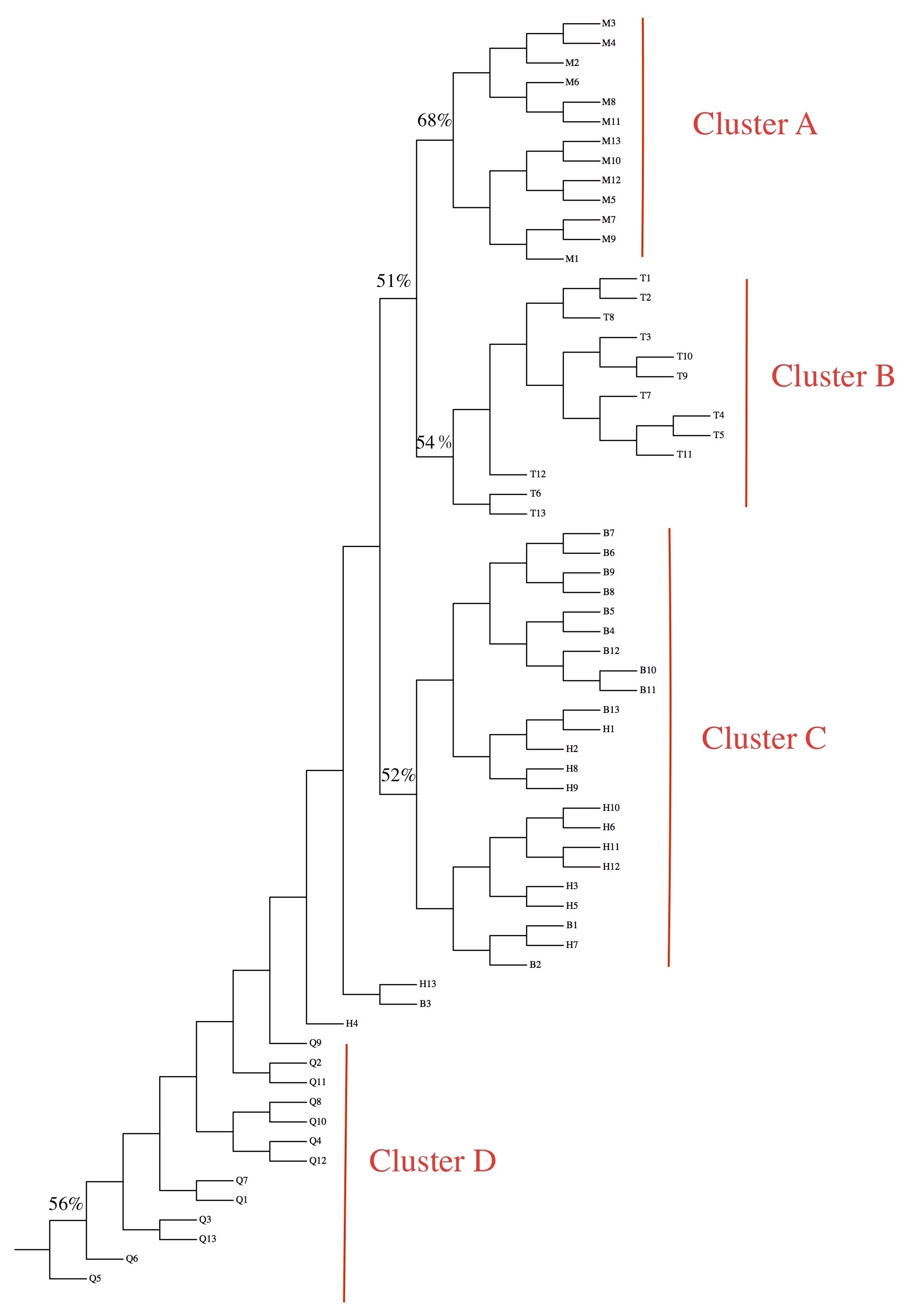

Supplement: S2 Fig — B, Hengdaohezi (Heilongjiang province) population (V. ramuliflora, 2x); H, Dailing (Heilongjiang province) population (V. ramuliflora, 2x); T, Qianshan (Liaoning province) population (V. ramuliflora, 2x); M, Changpai Mountains (Jilin province) population (V. ramuliflora, 4x); Q, Jiabei (Heilongjiang province) population (V. unijuga, 2x). The branches with bootstrap values of greater than 50% are marked. The numbers at the nodes indicate the percentage number of 1000 bootstrap replications. (JPG) [file pone.0170695.s004.jpg]
